# College Note

**Published:** 1902-11

**Authors:** 


### COLLEGE NOTE.

THE Medical Department of the University of Buffalo opened its course this year with the usual attendance, the freshmen class having the average number of matriculants.

A change has been made in the institution that has been very noticeable, "hazing" of freshmen and rushes between classes having been abolished. This is due to the decided action of the college faculty, which apparently has been successful. The upper class men, however, think that the incoming students will always miss something from their college experience, for they cannot look back with pleasant remembrances to their "introduction" to fellow students.

Students in physiological laboratory work are especially pleased with the outlook for study and investigation this year. During the last few weeks contractors have been at work constructing and fitting up a new laboratory for physiological research and experimental study. The necessary space has been obtained by extending the floor directly across the building from the anatomical rooms. This, of course, cuts down the size of the chemical laboratory, but in no way interferes with its efficiency. These improvements will afford increased facilities for individual work and research. New and thoroughly modern apparatus is being installed and prepared for use. Drs. Busch and Van Bergen will have charge of this department of medical instruction as heretofore.

